# Ant responses in a lycaenid–ant symbiosis are not facilitated by cuticular compounds alone

**DOI:** 10.1098/rsos.241320

**Published:** 2025-05-07

**Authors:** Dany S. Zemeitat, Marianne Coquilleau, Naomi E. Pierce, Mark A. Elgar

**Affiliations:** ^1^ BioSciences, The University of Melbourne, Melbourne, Victoria, Australia; ^2^ Museum of Comparative Zoology, Harvard University, Cambridge, MA, USA

**Keywords:** myrmecophily, colony recognition, cooperation, ants, Lycaenidae

## Abstract

Initiating partnerships in protective symbioses can be asymmetrical if there is a risk of attack from their symbionts. Myrmecophiles may encounter chemically mediated recognition systems that allow the host ants to distinguish nestmates from natural enemies, including non-nestmate conspecifics. The immature stages of the lycaenid butterfly *Jalmenus evagoras* form an obligate symbiosis with workers of *Iridomyrmex mayri* that protect them against natural enemies. However, the first instar larvae cannot anticipate this colony-specific chemical recognition system, since they are unlikely to encounter workers from the same colony that tended their mother. We show experimentally that workers of *I. mayri* can use chemical signals alone to distinguish between conspecifics and the larvae of *J. evagoras*; between nestmate and non-nestmate conspecifics and between larvae tended by nestmate and non-nestmate conspecifics. Nevertheless, we also show experimentally that while workers paid more attention to fourth than second instar larvae, they did not respond more aggressively to larvae that had been tended by non-nestmate versus nestmate workers. These data suggest that workers pay attention to other signals, perhaps via tactile, visual or vibratory sensory modalities, thereby allowing the butterfly myrmecophiles to mitigate the risks associated with the chemically mediated colony-specific recognition systems of their ant hosts.

## 1. Introduction

Communication is crucial for initiating cooperative relationships, where individuals must produce and receive signals to identify themselves as appropriate partners [1]. A key challenge for species that cooperate with ants is to overcome the chemically mediated defence of the host ant colony. Worker ants use chemical cues in the form of cuticular hydrocarbon (CHC) profiles or signature mixtures [2] to distinguish between nestmates and others, including non-nestmate conspecifics [3–6]. Nevertheless, taxonomically diverse insect myrmecophiles overcome these colony recognition cues to form symbiotic relationships ranging from mutualism to parasitism [7–12]. Accordingly, research has focused on comparing the CHCs and other cuticular compounds of myrmecophiles and their ant hosts, revealing variable degrees of resemblance [13–26].

While CHCs are thought to facilitate recognition between ants and their associates as they do between ant nestmates and non-nestmates, a complete match of the cuticular profile between myrmecophile and the host ant colony may not be necessary [19–21], and it is unclear how much variation is acceptable to meet resemblance thresholds. For example, certain classes of CHCs may be more important than others [27], and the specific signalling functions of CHCs can depend upon their location on the body of the ant, with the CHC profile differing between these body parts [28–30]. In other words, host ants may not pay attention to the entire cuticular chemical signature derived from whole-body extracts and instead use a sample of compounds. Further, recognition may be facilitated by signals other than CHCs, including other chemical compounds, or signals using other sensory modalities.

For myrmecophiles in which the adults disperse, colony-specific cuticular chemical resemblance to the host ants is unlikely before the myrmecophile has been ‘adopted’ by the host ant colony. This is because the new generation of myrmecophiles will not necessarily associate with the same ant colony as their parents—an adult female may disperse beyond the home range of her ‘natal’ ant colony and oviposit in locations inhabited by ants from neighbouring colonies and beyond. Thus, appeasement is crucial at the early developmental stage of the myrmecophile when it first contacts the ants and potentially each time it moults. Cuticular profiles of the myrmecophiles may change as they associate with the ants, either by feeding on the ant larvae [31] or by passively acquiring, through close contact with their host ants, a chemical profile that resembles the colony profile [32]. These possibilities highlight the importance of behavioural assays that can reveal the response of host ants to the cuticular chemistry of their myrmecophile associates [16,17,33,34], including at different stages of development.

The juvenile stages of many butterflies, especially within the Lycaenidae, form symbiotic associations with ants [11,12]. The relationships range from mutualism to parasitism, and while most are facultative where a few workers may attend the butterfly larvae, some are obligate, where the tending ants are critical for survival, and larvae are never found without their symbiotic ant partners [11,12,35]. Second instar and older larvae of many ant-tended species have specialized organs and glands [35–38] that provide sugar and amino acid food rewards [39–42], or additional substances that may appease, attract and/or manipulate workers [36,43–45]. First instar larvae typically lack some of these specialized organs [46] and small second instar larvae provide negligible food rewards. Nevertheless, these early instar larvae are often tended by ants, raising the question of how just-hatched lycaenid larvae reveal themselves as symbiotic partners with potential future benefits for the host ant colony.

The Australian Imperial Blue butterfly, *Jalmenus evagoras* (Donovan) (Lycaenidae), is distributed along the eastern Australian seaboard and obligately associated with several ant species, including *Iridomyrmex mayri* (Forel) [40] that provide protection from natural enemies [43], and in turn are rewarded with sugars, amino acids and potentially other, as yet unidentified compounds provided by the larvae [40,47]. (Note that in earlier publications, workers of this ant species were identified as *I. anceps*, but the assessment of workers from the same area, following a comprehensive revision of the genus *Iridomyrmex* [48], identified them as *I. mayri*). Three specialized organs, the Newcomer’s organ, tentacle organs and pore cupolae, all of which attract the attention of tending ants, are found on the second and older instar larvae of *J. evagoras* [46]. The first instar larvae lack Newcomer’s organ and tentacle organs and have only a few pore cupolae [46]. The larvae and pupae of *J. evagoras* aggregate, and the number of ants tending larvae is positively correlated with both larval instar and aggregation size: solitary first and second instar larvae are tended by only one or two workers at any time, while the morphologically similar fourth and fifth instars [46] are continuously tended by tens of workers [43]. The larvae typically remain on their natal host plant, pupating on the stem or branch. Volatile odours released by the tending ants are used by adult male and female butterflies as cues for locating mates [49] and oviposition sites [50] respectively, the latter reducing the time for tending ants to encounter recently hatched butterfly larvae. While adult females distinguish between different ant species and, to some extent, different conspecific ant populations, they readily oviposit on host plants infested with ants from colonies other than the one that tended them as juveniles [51]. Like many other ant–lycaenid symbioses, the role of cuticular chemical recognition signals that are necessary to establish and maintain the relationship with young larvae is poorly understood.

We used behavioural assays to investigate the role of cuticular chemicals as communication signals in the establishment and maintenance of the symbiosis between the lycaenid *J. evagoras* and its attendant ants *I. mayri*. First, we ask if chemical compounds alone allow workers of *I. mayri* (henceforth workers) to differentiate between larvae of *J. evagoras* (henceforth larvae) tended by nestmates compared with non-nestmates. We predict that workers exhibit more tending and fewer aggressive behaviours to cuticular extracts of fifth instar larvae that had been tended by nestmates versus non-nestmates. We also recorded the responses of workers towards the cuticular extracts of nestmate and non-nestmate conspecifics, expecting lower frequencies of aggression towards nestmates than non-nestmates, and lower frequencies of ‘tending’ behaviours towards extracts of workers than of larvae. In a second experiment, we explore whether workers respond similarly to young (second instar) and old (fourth instar) larvae that had been tended by nestmate or non-nestmate workers. If workers use chemical cues alone, then their responses should be broadly similar to those observed in the first experiment.

## 2. Methods

### 2.1. Response of workers to cuticular extracts

We collected workers of *I. mayri* and their associated fifth instar larvae of *J. evagoras* from ten *Acacia melanoxylon* host plants (representing ten different ant colonies) from field sites near Ebor, NSW during January and February 2016. Larval and ant cuticular extracts were obtained by immersing, respectively, three fifth instar larvae and 30 workers in hexane (2 ml, HPLC grade) for 10 min, and then transferring the eluate into clean vials. While this procedure introduces the possibility the eluate is contaminated by the animal defecating or regurgitating, we did not observe this behaviour nor is it likely that this contamination would generate a consistent bias in the response of workers.

Filter paper (Whatman, Grade 42) was cut into rectangular strips (10 mm × 7 mm) and placed into the cuticular solutions overnight. Filter paper soaked in hexane was used as a control. The filter paper was removed from the solution at least 2 h prior to the behavioural assay to ensure the hexane had evaporated. We used filter paper rectangles (henceforth strips), rather than smooth glass beads, because they provide a more efficient application of the eluate solutions over a greater surface area, have minimal visual cues, lower reflectivity and have proved effective in other experiments with ants (e.g. [3]). We refer to cuticular extracts, rather than CHC extracts, because cuticular washings with hexane may elute compounds other than just hydrocarbons, and these may stimulate ant behaviour.

For each trial, eight workers were collected from the *A. melanoxylon* host plant and placed into a petri dish (80 mm diameter by 15 mm height) that contained the experimental treatment. Workers were subjected to one of five cuticular extract treatments, where the filter paper strip had been immersed in the eluate of: (i) nestmate ant workers; (ii) non-nestmate ant workers; (iii) larvae of *J. evagoras* from the same host plant (and thus tended by nestmate ant workers); (iv) larvae of *J. evagoras* from another host plant with workers from a different colony; and (v) a hexane control. For each of the ten trees, we ran two trials for each of the five treatments.

We recorded behaviours of focal workers on a Sony Handycam AX53. These behaviours were commonly observed in the experiments and natural populations and appeared to be associated with cooperative or aggressive reactions (see table 1 for a description of these behaviours). We noted the total number of responses (table 1) of all eight focal workers towards the filter paper for 8 min. All trials took place at the field site, near the colony of the focal ant workers, thereby minimizing the time between worker collection and assay, which was typically less than an hour. Each filter paper strip was used in one trial only, and all trials involved different workers. Data from the video recordings were collected blind to the treatment.

**Table 1.** The responses of workers of *I. mayri* to cuticular extracts and live larvae. All six behavioural responses were recorded in the experiment involving cuticular extracts, while extensive antennating and biting only were recorded in the experiment involving second and fourth instar larvae of *J. evagoras*. Other infrequent behaviours (flexing and raising the gaster, remaining motionless on the filter paper) were not included in the analysis.

behavioural category	description of the behaviour of focal ant
brief antennations	The antenna is applied to the filter paper up to six times. This may represent an encounter behaviour that is not specifically associated with cooperative or aggressive behaviour
long antennations^a^	The antenna is applied to the filter paper/larva more than six times. This allows the worker to detect chemical cues, and thus may play a part in cooperative behaviour
biting^a^	Ants use their mandibles to bite the filter paper/larva. This behaviour is typically regarded as aggressive in interactions between non-nestmate conspecifics, but may have other functions in other contexts
grooming	Antennae are drawn across the first legs of the worker while standing on the filter paper. The behaviour may clean the antennae, allowing the worker to subsequently distinguish between familiar and unfamiliar conspecifics and symbionts
dragging	Filter paper is picked up by an ant using its mandibles and dragged/carried around the petri dish. This behaviour likely reflects tending behaviour

^a^ Only these behaviours were included in the analysis of the second experiment investigating the responses to the second and fourth instar larvae.

### 2.2. Response of workers to butterfly larvae

For the second experiment, we located eight *A. melanoxylon* host plants, each of which supported second and fourth instar larvae. Workers of *I. mayri* collected from these trees were subjected to one of four treatments in a block design with a single second or fourth instar larva of *J. evagoras* that had been collected from either the same or different *A. melanoxylon* host plant as the focal workers. Thus, the larvae had been tended by workers from the same or another colony, respectively. For convenience, we refer to nestmate or non-nestmate ants, and to larvae from the same or different host plants, as familiar or unfamiliar ants and larvae, respectively.

The behaviour of focal ants was recorded on a Sony Handycam AX53. We recorded the frequency of extensive antennating and biting larvae for 4 min. We confined our observations to these two behaviours, since other behaviours were very infrequent. All behavioural trials took place at the field site, near the colony of the focal ant workers, thereby minimizing the time between worker collection and assay, which was typically less than an hour. The variable number of larvae available from each tree meant that while all trees contributed at least one second and one fourth instar larva, some trees contributed more, yielding a total of 52 trials, with each larva used in one trial only, and all trials involving different ants. Data from the video recordings of all behavioural assays were collected blind to the treatment.

Ideally, we might compare similar young and old instar stages for both experiments, but logistically this was not possible. Obtaining cuticular eluates for the very small (2−3 mm) first or second instar larvae would require more individuals than could be readily collected from individual food plants. We assume that fourth and fifth instar larvae communicate similarly with attendant ants: they are morphologically extremely similar, possess the same traits that are associated with myrmecophily and differ primarily in size only [46]. Both are tended by large numbers of workers [43].

### 2.3. Statistical analyses

Data for the behavioural assays were analysed using the statistical package JMP® (v. 17.2, SAS Institute Inc., Cary, NC, 1989−2023). For each experiment, we used separate mixed models to investigate the variation in each behaviour, with species (*J. evagoras* and *I. mayri*) and familiarity (same tree or colony and different tree or colony) and their interaction term as fixed effects, and focal colony identity as a random effect. We used Tukey’s post hoc test with 95% confidence intervals to investigate significance levels between treatments when the species × familiarity interaction term was significant. Values are presented as rates per minute per eight ants.

## 3. Results

### 3.1. Response of workers to cuticular extracts

**Figure 1.**
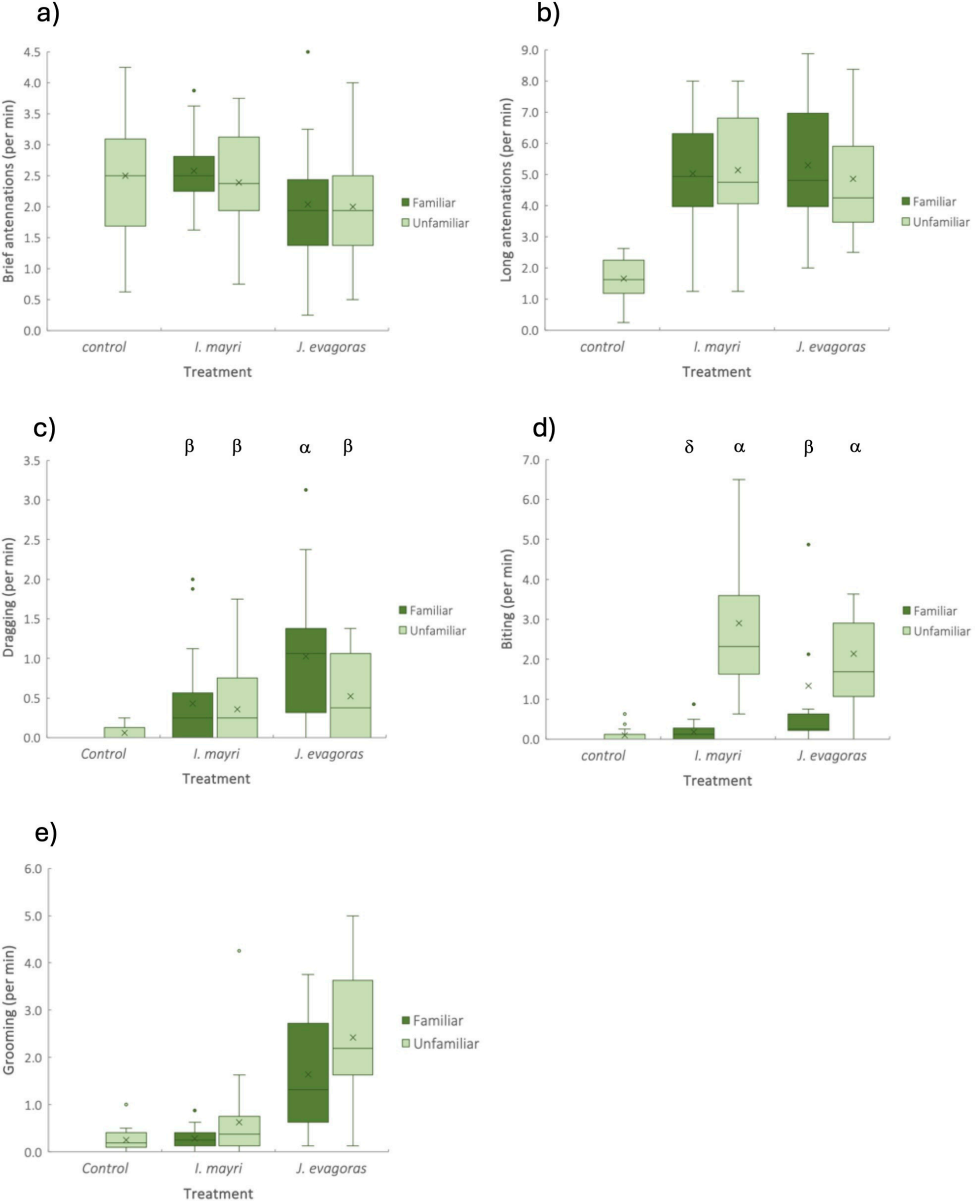
Behavioural responses of workers of *I. mayri* towards filter paper strips containing cuticular extracts from conspecific workers from the same colony (familiar) or different colony (unfamiliar); larvae of *J. evagoras* from the same *A. melanoxylon* host plant (familiar) or different *A. melanoxylon* host plant (unfamiliar); and a hexane control (designated unfamiliar). The behaviours, reported as frequency per minute per eight workers, include (a) brief antennations; (b) long antennations; (c) dragging; (d) biting (for convenience, two extreme outliers for ‘familiar larvae’ were removed for the figure only) and (e) grooming. The length of the box represents the interquartile range, with the line within the box representing the median and X representing the mean. The results of statistical analyses are given in table 2, and we used Tukey’s post hoc test (*p* < 0.05) where there was a significant species by familiarity interaction term (treatments that do not have the same Greek symbol are significantly different). Note the different *y*-axis scales.

Workers of *I. mayri* detect and respond to the cuticular extracts of both conspecific workers and the larvae of *J. evagoras* (table 2, figure 1). The frequency of long antennations by workers was three times greater on filter paper strips with cuticular extracts than control strips without cuticular extracts (figure 1b). Two behavioural responses differed according to the specific source of the cuticular odour. Brief antennations were less frequent on strips with extracts of larvae than extracts of workers, irrespective of their familiarity (F_1, 75_ = 8.90, *p* < 0.005; table 2a; figure 1a). In contrast, the frequency of long antennations was not explained by either species or treatment (table 2b; figure 1b). Aggressive biting behaviour was more frequently directed towards unfamiliar than familiar cuticular extracts, irrespective of whether the extract was derived from workers or larvae (F_1, 75_ = 103.13, *p* < 0.005; table 2d; figure 1d).

**Table 2.** Mixed models of variation in the behavioural responses of workers of *I. mayri* towards cuticular extracts of conspecifics and *J. evagoras* larvae collected from the same and different host trees/colonies, designated ‘familiar’ or ‘unfamiliar’, respectively. Note that the control treatment is not included in the statistical analysis.

	estimate	s.e.	d.f.	statistic	*p*
(a) brief antennations rate (*r^2^* = 0.23, *n* = 88)					
parameter estimates				*t* ratio	> |t|
intercept	2.26	0.12	8.5	19.51	<0.0001
species (*I. mayri)*	0.23	0.08	74.6	2.98	0.0039
treatment (unfamiliar)	−0.06	0.08	74.6	−0.75	0.45
species × treatment	−0.03	0.08	74.6	−0.46	0.65
REML variance component estimate				% total	Wald *p*
colony				11.9	0.27
fixed effects tests				*F* ratio	>*F*
main effects					
species (*I. mayri*, *J. evagoras*)			1,75	8.90	0.0039
treatment (unfamiliar, familiar)			1,75	0.56	0.45
interaction					
species × treatment			1,75	0.21	0.65
b) long antennations rate (*r^2^* = 0.27, *n* = 88)					
parameter estimates				*t* ratio	>|*t*|
intercept	5.15	0.32	9.1	15.78	<0.0001
species (*I. mayri*)	0.01	0.18	75.1	0.03	0.97
treatment (unfamiliar)	−0.08	0.18	75.1	−0.48	0.63
species × treatment	0.13	0.18	75.1	0.76	0.45
REML variance component estimate				% total	Wald *p*
colony				21.44	0.14
fixed effects tests				*F* ratio	>*F*
main effects					
species (*I. mayri*, *J. evagoras*)			1,75	0.00	0.97
treatment (unfamiliar, familiar)			1,75	0.23	0.63
interaction					
species × treatment			1,75	0.58	0.45
c) log (dragging rate+1) (*r^2^* = 0.50, *n* = 88)					
parameter estimates				*t* ratio	>|*t*|
intercept	0.17	0.03	8.8	5.29	0.0005
species (*I. mayri*)	−0.05	0.01	74.8	−3.72	0.0004
treatment (unfamiliar)	−0.03	0.01	74.8	−2.55	0.0127
species × treatment	0.02	0.01	74.8	1.97	0.0521
REML variance component estimate				% total	Wald *p*
colony				38.1	0.08
fixed effects tests				*F* ratio	>*F*
main effects					
species (*I. mayri*, *J. evagoras*)			1,75	13.82	0.0004
treatment (unfamiliar, familiar)			1,75	6.52	0.0127
interaction					
species x treatment			1,75	3.89	0.0521
d) log (biting rate+1) (*r^2^* = 0.74, *n* = 88)					
parameter estimates				*t* ratio	> |*t*|
intercept	0.33	0.05	8.9	7.13	<0.0001
species (*I. mayri*)	−0.02	0.02	74.9	−0.88	0.39
treatment (unfamiliar)	0.19	0.02	74.9	10.16	<0.0001
species x treatment	0.05	0.02	74.9	2.89	0.0051
REML variance component estimate				% total	Wald *p*
colony				36.9	0.08
fixed effects tests				*F* ratio	>*F*
main effects					
species (*I. mayri*, *J. evagoras*)			1,75	0.75	0.39
treatment (unfamiliar, familiar)			1,75	103.13	<0.0001
interaction					
species x treatment			1,75	8.33	0.0051
e) log (grooming rate+1) (*r^2^* = 0.58, *n* = 88)					
parameter estimates				*t* ratio	> |*t*|
intercept	0.29	0.02	9.5	12.88	<0.0001
species (*I. mayri)*	−0.15	0.02	75.7	−9.40	<0.0001
treatment (unfamiliar)	0.05	0.02	75.7	2.95	0.0042
species x treatment	−0.01	0.02	75.7	−0.74	0.46
REML variance component estimate				% total	Wald *p*
colony				8.8	0.32
fixed effects tests				*F* ratio	>*F*
main effects					
species (*I. mayri, J. evagoras)*			1, 76	86.41	<0.0001
treatment (unfamiliar, familiar)			1, 76	8.73	0.0042
interaction					
species x treatment			1, 76	0.54	0.46

Two behavioural responses of workers were influenced by both species and familiarity. The frequency of dragging was explained by a marginally significant species × treatment interaction term (F_1, 75_ = 3.89, *p* = 0.052; table 2c). Given the significance level, we conducted post hoc tests that revealed the behaviour was more frequently directed to strips with cuticular extracts from familiar larvae than to any other treatment (figure 1c). In contrast, workers groomed more frequently in the presence of cuticular extracts of larvae than of workers (F_1, 76_ = 86.41, *p* < 0.0001; table 2e) and if the extract was unfamiliar rather than familiar (F_1, 76_ = 8.73, *p* = 0.004; table 2e; figure 1e).

### 3.2. Response of workers to butterfly larvae

**Figure 2.**
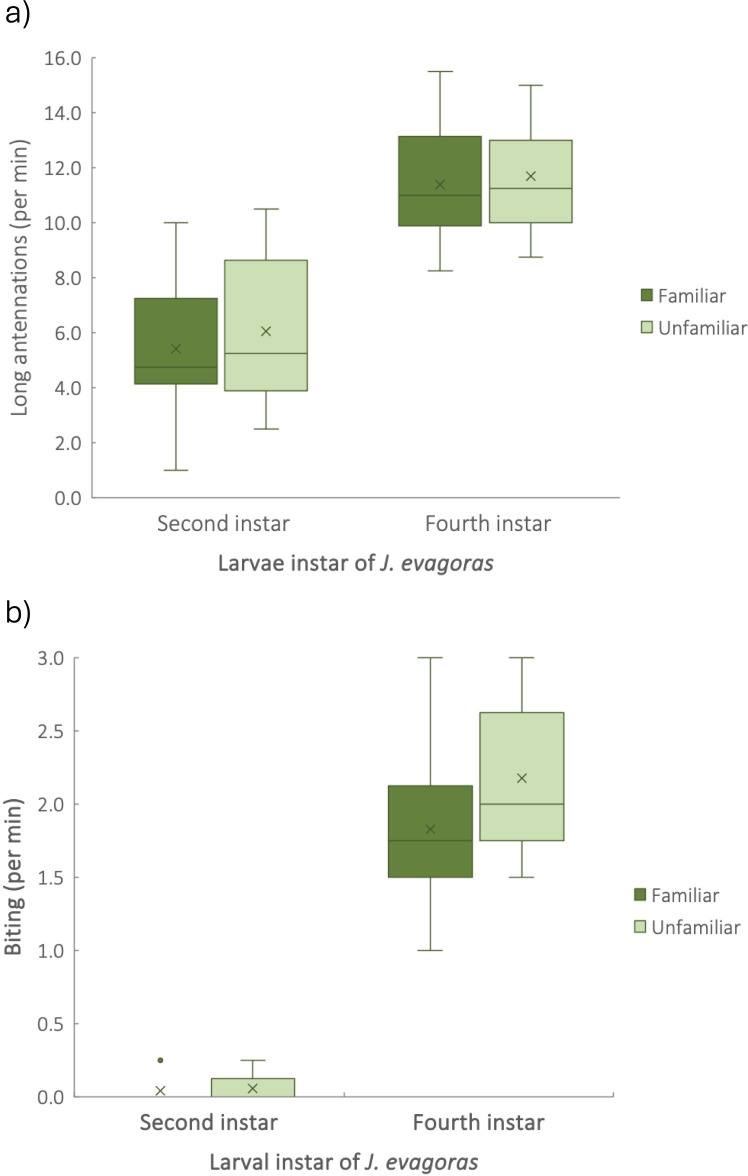
Behavioural responses of workers of *I. mayri* towards second and fourth instar larvae of *J. evagoras* from the same (familiar) or different (unfamiliar) *A. melanoxylon* host plant. Long antennations (a) and biting (b) are calculated as frequency per minute per eight workers. The length of the box represents the interquartile range, with the line within the box representing the median and X representing the mean. The results of statistical analyses are given in table 2. Note the different *y*-axis scales.

Extensive antennating and biting were the most frequent behaviours of workers in the presence of larvae, and so we confined our analysis to these two behaviours only. In general, workers paid more attention to older than younger larvae, with more frequent long antennations on the fourth than the second instar larvae (F_1, 41_ = 83.92, *p* < 0.0001; table 3a; figure 2a) irrespective of whether the larvae originated from the same or different host plants (F_1, 41_ = 0.53, *p* = 0.5; table 3a; figure 2a). Similarly, workers more frequently bit the fourth than second instar larvae (F_1, 39_ = 934.16, *p* < 0.0001; table 3b), with a marginally statistically significant difference in the frequency of biting familiar versus unfamiliar larvae (F_1, 39_ = 4.00, *p* = 0.049; table 3b) that was not supported by post hoc tests (figure 2b).

**Table 3.** Mixed models explaining variation in (a) extensive antennation and (b) biting behaviour of workers of *I. mayri* towards live second and fourth instar larvae of *J. evagoras* from the same (familiar) or different (unfamiliar) host tree.

	estimate	s.e.	d.f.	statistic	*p*
(a) long antennations rate (*r^2^* = 0.62, *n* = 52)					
parameter estimates				*t* ratio	> |*t*|
intercept	8.66	0.31	3.3	27.91	<0.0001
treatment (unfamiliar)	0.23	0.32	40.6	0.73	0.47
larval instar (fourth)	11.58	0.32	40.6	9.16	<0.0001
treatment × larval instar	−0.35	0.32	40.6	−0.27	0.79
REML variance component estimate				% total	Wald *p*
colony				0.0	0.63
fixed effects tests				*F* ratio	>*F*
main effects					
treatment (familiar, unfamiliar)			1,41	0.53	0.47
larval instar (second, fourth)			1,41	83.92	<0.0001
interaction					
treatment × larval instar			1,41	0.07	0.79
(b) log (biting rate+1) (*r^2^* = 0.96, *n* = 52)					
parameter estimates				*t ratio*	>|*t*|
intercept	0.24	0.01	5.2	21.24	<0.0001
treatment (unfamiliar)	0.01	0.01	39.3	2.03	0.0491
larvae (fourth instar)	0.22	0.01	39.3	30.56	<0.0001
treatment × larval instar	0.01	0.01	39.3	1.53	0.13
REML variance component estimate				% total	Wald *p*
colony				16.04	0.39
fixed effects tests				*F* ratio	>*F*
main effects					
treatment (familiar, unfamiliar)			1,39	4.12	0.0491
larvae (second, fourth instar)			1,39	934.16	<0.0001
interaction					
treatment × larval instar			1,39	2.33	0.13

## 4. Discussion

Our experiments reveal three lines of evidence that the symbiosis between lycaenid butterfly larvae and their attendant ants is facilitated by chemical signals. First, workers of *I. mayri* typically paid far more attention to filter paper strips with chemicals from the cuticle of fifth instar larvae of *J. evagoras* than to control strips and reacted less aggressively towards the cuticular extracts of larvae that had associated with nestmate versus non-nestmate workers. The workers were also less aggressive to strips with the cuticular extracts of nestmate versus non-nestmate conspecifics. Second, workers spent more time dragging filter paper strips containing extracts from larvae collected from the same host plant than any other treatment. Third, workers spent more time grooming after encountering filter paper strips with cuticular extracts from larvae versus conspecifics. The patterns of dragging and grooming behaviour suggest that workers can distinguish between conspecifics and butterfly larvae using chemical cues only. Finally, while workers paid more attention to older than younger larvae, they did not appear to distinguish between familiar and unfamiliar larvae, suggesting that workers may pay attention to other chemical cues or other sensory modalities. The similar frequency of brief antennations directed towards control and other treatment strips contrasts with the relatively low frequencies of the other four behaviours, suggesting that brief antennations represent initial encounter behaviour.

The adoption strategies of early instar larvae of *J. evagoras* by their attendant ants may not include colony-specific cuticular compounds, which are widely thought to facilitate myrmecophile symbioses as they allow ant workers to distinguish between nestmates and others [3–6]. For example, larvae of the parasitic Alcon blue butterfly *Maculinea rebeli* synthesize a subset of the ant species-specific CHCs prior to adoption by the host ant, followed by the acquisition of additional colony-specific blends after adoption into the ant nest [16]. Perhaps workers of *I. mayri* adopt early instar larvae independent of their colony of origin by recognizing a general ‘larval’ cuticular odour that stimulates tending rather than aggressive behaviour (see also [45])—workers did not distinguish between familiar and unfamiliar second instar larvae and a similar pattern is likely for first instar larvae, since they are even less frequently tended by ants [43]. Further, workers spent more time dragging strips with the cuticular extract of larvae than of conspecific workers. Such an adoption strategy by early instars of *J. evagoras* would be adaptive because adult females do not oviposit exclusively on food trees located within the home range of the colony of ants that tended them during their juvenile stages. The larger retinue of workers tending older larvae may arise from qualitative or quantitative changes in cuticular compounds acquired during development, or differences in the opportunity for tending ants to learn, either individually [52,53] or collectively [54], the odour profiles of the older larvae that provide food rewards.

While it is unlikely that cuticular compounds play no role in facilitating the association between the larvae of *J. evagoras* and their attendant *I. mayri* ants, as has been suggested for social parasites of ants [9], several lines of evidence suggest the involvement of other signalling modalities. First, there were differences between the response of workers to filter paper strips, which provide chemical cues only, and larvae, which may provide additional cues, including those perceived through other sensory modalities. Workers frequently dragged strips containing familiar fifth instar cuticular extract but rarely exhibited similar behaviour towards second or fourth instar larvae, suggesting that dragging behaviour caused by chemical signals is subsequently modified by the physical presence of the larvae. Second, the more aggressive responses of workers to the cuticular extract of unfamiliar than familiar fifth instar larvae were not apparent when they were presented with second and fourth instar larvae—workers were similarly aggressive to familiar and unfamiliar larvae, irrespective of the instar. Perhaps workers are unable to distinguish between familiar and unfamiliar second instar larvae, and their behaviour towards fourth instar larvae is influenced by the production of vibratory cues [55], or the provision of significant food rewards [40], neither of which are available to younger larvae. It is not clear why workers bit fourth instar larvae more frequently than second instar larvae. It may simply reflect differences in opportunity afforded by the larger body, or the functional significance of the behaviour may be more nuanced and context dependent—reflecting an aggressive response when workers encounter chemical cues only but functioning in a different way when workers encounter larvae.

While the difference in worker response to fourth instar larvae and fifth instar cuticular extract might reflect differences in the cuticular profiles of fourth and fifth instar larvae, there are several reasons why this seems unlikely: fourth and fifth instar larvae are morphologically very similar, heavily tended by ants, and rarely, if ever, move between host plants. Moreover, it is not clear why such differences might exist between fourth and fifth instar larvae, but not between second and fourth instar larvae. While changes in cuticular chemical profiles during larval development have been documented in beetles [56] and flies [57,58], but not wasps [59], any differences across larval development in *J. evagoras* may not necessarily cause changes in ant responses. Theory holds that traits, including volatile and cuticular chemical compounds, may evolve a signalling function if they provide reliable information to the receiver [60,61], and so workers should only pay attention to those cuticular compounds that are informative. Interestingly, host ants *Camponotus japonicus* of the parasitic lycaenid *Niphanda fusca* react differently to second and third instar larvae that are morphologically different but have similar CHC profiles, suggesting they respond to other cues [62].

Larvae of *J. evagoras* may acquire colony-specific cuticular odours from their tending ants, as reported for beetles [13], wasps [63], spiders [31] and other parasitic lycaenids [23]. The similar frequency with which workers bit strips with the cuticular extract of unfamiliar larvae and non-nestmate conspecifics is consistent with this view. Nevertheless, acquiring colony-specific odours may be a consequence of ant attention, rather than a means of facilitating the symbiosis, leaving untended first instar and recently moulted larvae vulnerable. First instar larvae may find refuge in joining groups of larvae, and field surveys indicate that early instar larvae are more likely to aggregate than older larvae [43], but this strategy is available only when the food plant supports larvae of different instars. Our assays with live larvae revealed that ant attention was influenced by age but not familiarity, suggesting that tending ants may pay less attention to colony-specific cuticular profiles in the presence of other signals or cues, including via other sensory modalities [55]. Clearly, we require a more nuanced alignment of behavioural assays and analyses of the chemical profiles of myrmecophile larvae.

Investigations of chemical communication between myrmecophiles and their ant hosts emphasize the significance of colony recognition by focusing on the degree of chemical similarity of cuticular compounds [13–26], and the level of aggressive response of symbiotic ants. Our behavioural assays highlight that the lycaenid cuticular profile contains chemicals that distinguish it from that of attendant ants and thus elicits different responses beyond biting behaviour. For example, some behavioural responses of workers, including dragging and grooming, differed between the cuticular extracts of lycaenid larvae versus those of conspecific ants. Moreover, workers also spent significantly more time ‘dragging’ filter paper with cuticular extracts from larvae that had been tended by nestmates than those either from larvae tended by non-nestmates or from conspecific workers. This behaviour is likely to be protective rather than aggressive: workers under natural conditions often drag or carry early instar larvae and especially do so if attempts are made to remove larvae with forceps—unsuccessful removal typically results in the workers carrying the larvae away. Perhaps similar chemical cues are involved in the adoption process of other lycaenid mutualists, whose larvae are taken into the nests of their host ants, as is the case for the larvae of parasitic *Phengaris* (= *Maculinea*) lycaenids [16,64].

## Data Availability

The datasets supporting this article have been made available at Dryad [65].
